# The Influence of Temperature on the Antiviral Response of mIgM^+^ B Lymphocytes Against *Hirame Novirhabdovirus* in Flounder (*Paralichthys olivaceus*)

**DOI:** 10.3389/fimmu.2022.802638

**Published:** 2022-02-07

**Authors:** Xiaoqian Tang, Xinbiao Ma, Jing Cao, Xiuzhen Sheng, Jing Xing, Heng Chi, Wenbin Zhan

**Affiliations:** ^1^ Laboratory of Pathology and Immunology of Aquatic Animals, Key Laboratory of Mariculture, Ministry of Education (KLMME), Ocean University of China, Qingdao, China; ^2^ Laboratory for Marine Fisheries Science and Food Production Processes, Qingdao National Laboratory for Marine Science and Technology, Qingdao, China

**Keywords:** *Hirame novirhabdovirus*, mIgM^+^ B lymphocytes, *Paralichthys olivaceus*, antiviral immune response, temperature

## Abstract

*Hirame novirhabdovirus* (HIRRV) is an ongoing threat to the aquaculture industry. The water temperature for the onset of HIRRV is below 15°C, the peak is about 10°C, but no mortality is observed over 20°C. Previous studies found the positive signal of matrix protein of HIRRV (HIRRV-M) was detected in the peripheral blood leukocytes of viral-infected flounder. Flow cytometry and indirect immunofluorescence assay showed that HIRRV-M was detected in mIgM^+^ B lymphocytes in viral-infected flounder maintained at 10°C and 20°C, and 22% mIgM^+^ B lymphocytes are infected at 10°C while 13% are infected at 20°C, indicating that HIRRV could invade into mIgM^+^ B lymphocytes. Absolute quantitative RT-PCR showed that the viral copies in mIgM^+^ B lymphocytes were significantly increased at 24 h post infection (hpi) both at 10°C and 20°C, but the viral copies in 10°C infection group were significantly higher than that in 20°C infection group at 72 hpi and 96 hpi. Furthermore, the B lymphocytes were sorted from HIRRV-infected flounder maintained at 10°C and 20°C for RNA-seq. The results showed that the differentially expression genes in mIgM^+^ B lymphocyte of healthy flounder at 10°C and 20°C were mainly enriched in metabolic pathways. Lipid metabolism and Amino acid metabolism were enhanced at 10°C, while Glucose metabolism was enhanced at 20°C. In contrast, HIRRV infection at 10°C induced the up-regulation of the Complement and coagulation cascades, FcγR-mediated phagocytosis, Platelets activation, Leukocyte transendothelial migration and Natural killer cell mediated cytotoxicity pathways at 72 hpi. HIRRV infection at 20°C induced the up-regulation of the Antigen processing and presentation pathway at 72 hpi. Subsequently, the temporal expression patterns of 16 genes involved in Antigen processing and presentation pathway were investigated by qRT-PCR, and results showed that the pathway was significantly activated by HIRRV infection at 20°C but inhibited at 10°C. In conclusion, HIRRV could invade into mIgM^+^ B lymphocytes and elicit differential immune response under 10°C and 20°C, which provide a deep insight into the antiviral response in mIgM^+^ B lymphocytes.

## Introduction

*Hirame novirhabdovirus* (HIRRV) is one of the most important etiological agents in fish aquaculture industry (1, 2). HIRRV belongs to the *Novirhabdovirus* genus and its genome is a single-stranded, negative-sense RNA encoding six viral proteins including nucleoprotein (N), phosphoprotein (P), matrix protein (M), glycoprotein (G), non-structural protein (NV) and RNA polymerase (L) (3). HIRRV has a broad host range including marine and freshwater fish such as flounder (*Paralichthys olivaceu*) (4), black seabream (*Acanthopagrus schlegeli*) (5), grayling (*Thymallus thymallus*) and brown trout (*Salmo trutta*) (6). The incidence of HIRRV infection is closely related to the water temperature, with a peak occurrence in winter and early spring. High mortality due to HIRRV infection occurred at 10°C, but no mortality was observed at above 20°C (7). Similar phenomenons were also found in other cultured fish infected with viral haemorrhagic septicaemia virus (VHSV) (8), spring viraemia of carp (SVCV) (9), infectious spleen and kidney necrosis virus (ISKNV) (10) and koi herpesvirus (KHV) (11). As poikilothermic vertebrates, water temperature rapidly and significantly affects fish body temperature, which has great impact on physiological processes and immune functions (12, 13). Previous studies revealed that acute and chronic exposure to suboptimal temperature would yield suppressive effects on the innate and adaptive immune systems of fishes, particularly on adaptive immunity (14). In general, low temperature would lead to a shutdown or inhibition of fish immune response. For instance, early studies in channel catfish (*Ictalurus punctatus*) showed that the the proliferation and immune response of T and B cells were significantly inhibited while maintaining at low temperatures (15–17). Similarly, the expression of B lymphocyte marker genes including *IgM*, *IgT*, Paired Box 5 (*Pax5*), and B lymphocyte maturation-induced protein-1 (*Blimp1*) in rainbow trout (*Oncorhynchus mykiss*) were strongly up-regulated at 15°C relative to 12°C, suggesting that B lymphocyte proliferation may be impaired under low temperature (18). Inhibitory effects on adaptive immune response at low temperatures have also been observed in common carp, T lymphocyte proliferation in PBLs was proportional to temperature with proliferation increasing from 12°C to 28°C both *in vivo* and *in vitro* (19). The antigen presentation and antibody production in *Paralichthys olivaceus* and rainbow trout were also revealed to be temperature-dependant, exhibiting a negative impact induced by suboptimal temperature exposure (20, 21). These data seem to suggest that low temperatures negatively impact B lymphocyte and T lymphocyte proliferation in fish, which may adversely affect their ability to limit the spread of infection and mediate specific responses against pathogens. In contrast, an enhancement of killing activity of cytotoxic cells was found in the carp (*Cyprinus carpio*), maintained at low temperature (22). Similarly, blood granulocytes from tench (*Tinca tinca* L.) maintained at low temperatures displayed a greater phagocytic capacity and production of superoxide anions than that at high temperatures (23), suggesting the innate immune system of fish has a greater adaptability to the low temperatures.

B lymphocytes are known as an important professional antigen presenting cells (APC) type involved in the activation of helper T (Th) cell (24–26), and B cells of teleost even possess the ability to internalize and present particulate antigens (25, 27). The researches on the fish rhabdovirus showed that mIgM^+^ B lymphocytes are the main cell subset infiltrating in the intramuscular injection area post vaccination with VHSV DNA vaccine (28). Similarly, after intraperitoneal injection of inactivated VHSV, mIgM^+^ B lymphocytes also constituted the main leukocyte subset in the abdominal cavity of rainbow trout (*Oncorhynchus mykiss*) (29), indicating that mIgM^+^ B lymphocytes play a potential role in the initial recognition and processing of viral antigens and it is essential for inducing adaptive immunity. As the key driver of humoral immune responses, B cells play a unique part through their production of antibodies that can both neutralize and clear viral particles before virus entry into a host cell. Protective antibodies are produced even before first exposure to the pathogen, through the regulated secretion of so-called natural antibodies. Previous research in rainbow trout also found that natural antibodies secreted by B lymphocytes was shown to take part in the immune defense against viral infection (30). Moreover, B cells can regulate innate immunity by secreting a variety of cytokines (31). One B cell subset with the ability to produce IFN-γ were identified in human and mice, which can suppress the infection of intracellular pathogens (32, 33). In teleost, mIgM^+^ B lymphocytes have a strong ability to sense virus ligands, the mIgM^+^ B lymphocytes from different tissues of rainbow trout express toll like receptors (*TLRs*) including *TLR3*, *7*, *8* and *9*, which were known to recognize highly conserved structures of viral origin (34). It was well documented mammalian *TLR3* is the main *TLR* responsible for sensing dsRNA virus and sends signals through TRIF pathway together with *TLR4* to induce the production of *IFN-β* (35). Orthologs of mammalian *TLR3* have been identified in *Paralichthys olivaceus*, and it is proved that extracellular and intracellular polycytidylic acid (poly I:C) cause *TLR3* to activate both IFN and NF-κB pathways (36). Recent study has found that VHSV could enter mIgM^+^ B lymphocytes and initiate viral transcription, suggesting that rhabdoviruses have the capacity to infect mIgM^+^ B lymphocytes (37). Previously, we also found that HIRRV could infect a portion of leukocytes in viral infected flounder (38), suggesting mIgM^+^ B lymphocytes may be the target cells of HIRRV. However, the interaction between HIRRV and mIgM^+^ B lymphocytes remains unclear.

Thus, in the present work, the infection characteristics of HIRRV to mIgM^+^ B lymphocytes were examined by IIFA and flow cytometry. In order to further explore potential role of mIgM^+^ B lymphocytes in HIRRV infection, the mIgM^+^ B lymphocytes of HIRRV-infected flounder under different water temperatures were separated by immunomagnetic bead (IMB) method and submitted for RNA-Seq, and the mechanism of antiviral response in mIgM^+^ B lymphocytes at different temperatures was analyzed, so as to provide improved understanding of antiviral functions of mIgM^+^ B lymphocytes.

## Materials and Methods

### Experimental Fish and Virus

Healthy flounders (*Paralichthys olivaceus*, 750 ± 50 g) were obtained from a fish farm (Rizhao, China) and confirmed free from HIRRV infection by nested PCR. The fish were raised in a circulating-water temperature-controlled aquaculture system at 15°C, then divided into two groups. The water temperatures of two groups were gradually adjusted to 10°C and 20°C within 3 days, respectively, and then allowed for 2 weeks acclimatization. HIRRV strain CNPo2015 was previously isolated from the naturally infected flounder and stored at -80°C (39). The virus stain was inoculated to a monolayer of Epithelioma papulosum cyprini (EPC) cells cultured in M199 medium (Gibco) with 2% fetal bovine serum (Gibco) and antibiotics at 15°C. When the cytopathic effect (CPE) was extensive, the cellular supernatants were collected and centrifuged to remove cell debris. The titer of virus was determined and adjusted to 1.0 × 10^7^ TCID_50/_mL.

### Experimental Infection and Sampling

The flounder raised under two different temperatures (10°C and 20°C) were intraperitoneally injected with 800 μl of virus suspension (1.0 × 10^7^ TCID_50_), and the control fish were injected with the same volume of PBS. For RNA-Seq, mIgM^+^ B lymphocytes sorted from peripheral blood leukocytes (PBLs) were collected from three fish of each group at 0, 24,and 72 hour post infection (hpi) and sharp-frozen in liquid nitrogen. For qRT-PCR, mIgM^+^ B lymphocytes sorted from PBLs were collected from three fish in each group at 0, 24, 48, 72 and 96 hpi, and the samples were immersed in RNAlater (Takara) and then stored at -80°C. Before injection, the flounders were anaesthetized with 100 mg/ml MS-222 (Sigma). The flounders used in this study was carried out strictly in line with procedures in the Guide for the Use of Experimental Animals of Ocean University of China.

### Cell Sorting

Leukocytes were isolated from the peripheral blood of three individual flounders by discontinuous Percoll gradient centrifugation according to previous method (38). Briefly, blood was drawn from the caudal vein and diluted 1:1 in solution (65% RPMI-1640 containing 20 IU ml^−1^ heparin, 0.1% w/v NaN_3_ and 1% w/v BSA), and centrifuged at 100 g for 10 min. After centrifugation to get rid of the red blood cells, the supernatant were layered over a discontinuous gradient of Percoll with 1.020- 1.070 g/cm^3^ densities and centrifuged at 840 g for 30 min. The cell layers at the interface were collected and washed 3 times with PBS containing 5% (v/v) new-born calf serum by centrifugation at 640 g, then suspended in PBS. The PBLs were adjusted to 1.0 × 10^6^ cells/ml for sorting of the mIgM^+^ B lymphocytes using IMB method. Briefly, the isolated PBLs from each fish were pooled into one sample and incubated with the the specific monoclonal antibody (mAb) against flounder IgM, which was produced previously by our lab (40). The cells were then washed three times using magnetic activated cell sorting (MACS) buffer (PBS containing 2 mM EDTA and 0.5% bovine serum albumin). The leukocyte pellets were resuspended in MACS buffer (80 μl per 10^7^ cells) and incubated with goat anti-mouse IgG magnetic beads (20 μl per 10^7^ cells, Miltenyi Biotec) for 15 min at 4°C. After another washing step, the leukocytes were resuspended in MACS buffer (500 μl per 10^8^ cells) and filtered through a 40 μm nylon mesh. A MACS LS column (Miltenyi Biotech) was installed in a MACS separator according to the manufacturer’s instructions and balanced with 3 ml MACS buffer. The leukocyte suspension was then passed through the column, and magnetically unlabeled leukocytes were washed off using MACS buffer. The MACS LS column was subsequently removed from the MACS separator, and the magnetically labeled leukocytes were collected. To obtain a high-purity mIgM^+^ B lymphocytes, the magnetically labeled leukocytes were sorted for a second time using a new balanced MACS LS column. The purity of sorted mIgM^+^ B lymphocytes was examined by flow cytometry and IIFA. A trypan blue (0.4%) exclusion assay was performed to determine the viability of the sorted cells. A primary mAb specific for flounder mIgM and a secondary Alexa Fluor^®^ 488-conjugated goat anti-mouse IgG antibody (Invitrogen) were used for flow cytometry and IIFA.

### Detection of HIRRV in mIgM^+^ B Lymphocytes

In order to investigate the infection characteristics of HIRRV to mIgM^+^ B lymphocytes, the mAb against matrix protein of HIRRV (HIRRV-M) produced previously by our lab was employed to detect the HIRRV host cells (38). For flow cytometric analysis, the PBLs isolated at 24 hpi under two different temperatures (1.0 × 10^6^ cells/ml) were incubated with the mAb against flounder IgM for 1 h at 37°C. After washed three times with PBS, the leukocytes were suspended and incubated with Alexa Fluor^®^ 488-conjugated goat anti-mouse IgG antibody and Alexa Fluor^®^ 649 labeled mouse anti-HIRRV-M mAb (1:1000) for 45 min at 37°C in dark. After washed as above, the PBLs suspensions were analyzed using BD Accuri™ C6 flow cytometer (BD Biosciences). For IIFA, the PBLs were counterstained with DAPI (Bio-Legend) for 10 min at 37°C in dark. After the last washing, 20 μl PBLs suspension (1 × 10^6^ cells/ml) was dripped onto APES coated slides, and the PBLs were settled and fixed onto slides after 2 h, and then observed under a fluorescence microscope (Olympus DP70).

### Quantitative Real-Time PCR

In order to determine the replication of HIRRV in mIgM^+^ B lymphocytes, 1 μg total RNA extracted from mIgM^+^ B lymphocytes was reversely transcribed into cDNA in a 20 μL reaction. Then, 2 μL cDNA was used as the templates, and a pair of specific primers (F:5’-CTTCCTGATTGTGATGTCTGCG-3’and R:5’-CAACGATACTCC TGTGATTCCG-3’) were designed for PCR amplification of the fragment of HIRRV glycoprotein (G) gene. Each sample was run in triplicate, and the non-infected samples were used as the negative control. After amplification, melting curve analysis was performed to ensure no nonspecific amplification. Finally, viral copy numbers were determined by extrapolating Ct values from the standard curve which was established previously (4). The data was expressed as mean log_10_ copies/0.1 μg RNA.

Specific primers of immune-related genes were designed according the sequencing results with Primer Premier 5, 18S rRNA of flounder was used as the internal reference to normalize the expression level. The qRT-PCR was performed using SYBR GreenIMaster (ABM) in LightCycler^®^ 480 II Real Time System (Roche), and the expression levels of selected genes were analyzed by the 2^−△△Ct^ method, the primers used in this part were listed in **Table 1**.

**Table 1:** Primers for quantitative PCR analysis.

Primers	Sequences (5′ -3′)	Length (bp)	Accession No.	Amplification efficiency
Tap1	F: CATAACCCTGTAACACCCTTT	246	NW_017863985	99.25%
	R: TCTTCATCTCCCATCCACTCC			
Tap2	F: ATCTGCGTTGACCTTCTTTGC	319	XM_020113520	98.44%
	R: GAATGTCACCTTCTCGTACCCTT			
Rfx5	F: GATGGGCGTTTCTCCTTTAGC	272	NW_017865372	98.72%
	R: CAAGTTTCGGTTCCTTTCACT			
β2M	F: TTTCCATCCTTTAGCAGCGTA	102	XM_020100021	96.84%
	R: CACCCGTGAGCCAGAAGAGTT			
Li	F: CATACTCTTGCGAACACCTTT	300	XM_020081381	98.84%
	R: CTGATGGATTTCACTTTGGAT			
NFYA	F: TCTTTGGGGATCTTGCCCTCT	256	XM_020089478	99.26%
	R: ACACTAACACCACGAACAGCG			
NFYB	F: GTGGGAATCTGTTGATATGAGGT	134	XM_020099782	99.47%
	R: GTGGGAATCTGTTGATATGAGGT			
NFYC	F: CTGTCTGATTGGTGGACTGAATG	218	XM_020090802	97.42%
	R: CCGTCTAGCTCAGCCTGTCTC			
Hsp70	F: CAACCTCACGGGTAACAACTA	184	XM_020089177	100.36%
	R: CATCTGGGAATCCTCTGCTGG			
HSP90a.1	F: CAAAGAGCAAGGCACGAAACT	160	XM_020097585	93.30%
	R: GTTCATCGGCTACCCTATTACA			
MHC I	F: AGACCACAGGCTGTTATCACCA	301	XM_020108465	94.99%
	R: TCTTCCCATGCTCCACGAA			
ERp57	F: ACAGGGACGGAACTTATCAACG	208	XM_020106772	96.97%
	R: TCATCGGACTGGAGGGAGG			
Ciita	F: CCTCTGTCTTTCGTCCCATCT	328	XM_020103040	95.57%
	R: TTCATACTCGCTCAAGCAACC			
Canx	F: AGTGTTGCGGTCGTTGGTGAT	284	XM_020110256	99.47%
	R: GAGGACATGGACGGTGAATGG			
TNF	F: TGATAAGGCTTCCGTTTCTGT	285	XM_020094436	93.34%
	R: TGCTGTTCCTGCTGGTGAGTC			
CALR	F: GGCCACAAATATCGGGACCAA	208	XM_020081423	94.61%
	R: TCAGGACGCTCGCTTCTACGC			
TLR9	F: TTGCCACTGAACCGACCTTT	222	XM_020096317.1	97.09%
	R: CAGTCTACCGTGGTGGCATT			
TLR7	F: TGCCCAAATAATTCCCCGCT	131	XM_020089659.1	101.94%
	R: AGTCCTGTTGTGGTGGCAAA			
CCL20	F: GTGAGTTACAGCAAGCGTCC	157	XM_020103201.1	96.02%
	R: TCATCCCTCTCTGGGTCCATT			
TNFRSF10B	F: TGTAAACTGGAAGAGGAGCACA	134	XM_020090528.1	98.63%
	R: TTGTCGCTGATCCCAAGGTG			
CAPS3	F: GCTGAGTCATGGAGACGAGG	113	XM_020090528.1	101.01%
	F: TCGGTTTGCCAACCAGACTT			
TGFB1	F: AAACAGAGGACGAGCGTTGT	245	XM_020088612.1	96.47%
	R: TGTACATGCATCCTTCGCGT			
IL12B	F: TCAGTTTGAGCTGTCGCACT	208	XM_020099047.1	99.03%
	R: GCTGGATGGAGGATCGATGG			
IgM	F: CTATACCAACTCTGTTCCCTG	224	AF226284.1	97.96%
	R: GCTATTGTCCCACTCCTGT			
18S rRNA	F: GGTCGTGATGCCCTTAGATGTC	102	EF_126037	97.71%
	R: AGTGGGGTTCAGCGGGTTAC			

### Detection of Apoptotic Lymphocytes by Flow Cytometry

Here we adopted Annexin V-FITC (Vazyme) method combine with flow cytometry to quantitatively detect the apoptotic lymphocytes. Lymphocytes were isolated at 0, 24 and 72 h from HIRRV infected flounders at 10°C and 20°C. All procedures were conducted following the instructions of manufacturer. Briefly, approximately 10^5^ lymphocytes were incubated with the specific monoclonal antibody (mAb) against flounders IgM (1:1000 diluted in PBS) for 1 h at 37°C, which was produced previously by our lab (40). After washing three times with PBS, the lymphocytes were suspended and incubated with Dylight 649-conjugated goat anti-mouse IgG antibody (1:1000 diluted in PBS) for 45 min at 37°C in dark. After washed as above, the lymphocytes were suspended in 100 μl binding buffer, and 5 μl Annexin V-FITC was added and incubated for 10 min at room temperature in dark, then 400 μl of binding buffer was added. Annexin V-FITC positive lymphocytes were analyzed using BD FACS Aira Fusion flow cytometer (BD Biosciences).

### cDNA Library Construction and Sequencing

The total RNA was extracted from the sorted mIgM^+^ B lymphocytes using Trizol reagent kit (Invitrogen) according to the manufacturer’s protocol. RNA quality was determined on a NanoDrop 8000 spectrophotometer (Thermo Scientific) and checked using RNase free agarose gel electrophoresis. After evaluating the quality of the total RNA, the eukaryotic mRNA was enriched by Oligo (dT) beads (Thermo Fisher). Then, the enriched mRNA was fragmented into short fragments using fragmentation buffer and reverse transcripted into cDNA with random primers. Second-strand cDNA were synthesized by DNA polymerase I, RNase H, dNTP and buffer. Then the cDNA fragments were purified with QiaQuick PCR extraction kit (Qiagen), end repaired, poly(A) added, and ligated to Illumina sequencing adapters. The ligation products were size selected by agarose gel electrophoresis, PCR amplified, and sequenced using Illumina HiSeq™ 2500 by Gene Denovo Biotechnology Co. (Guangzhou, China).

### Gene Annotation and Differential Expression Analysis

The genome was obtained from the US National Center for Biotechology Information database (https://www.ncbi.nlm.nih.gov/genome/?term=Paralichthys%20olivaceus). After sequencing, clean reads were obtained to trim the raw data by removing adaptor and low-quality sequences (Q < 20), ambiguous nucleotides, and short reads (<30 bp), using the CLC Genomics Workbench (CLC bio, Denmark). Clean reads from the treatment and control groups were next aligned to the flounder genome using the TopHat bioinformatics tool. Based on the beta negative binomial distribution model, the Cuffquant and Cuffnorm components of Cufflinks software were used to quantify the expression level of transcripts and genes through the positional information of the reads mapped to the reference genome. Fragments Per Kilobase of transcript per Million fragments mapped (FPKM) was used as an indicator of transcript or gene expression levels. Differential expression analysis of mIgM^+^ B lymphocytes was performed by DESeq2 software between two different groups. The genes/transcripts with the parameter of false discovery rate (FDR) below 0.05 and absolute fold change ≥2 were considered differentially expressed genes/transcripts. Gene Set Enrichment Analysis (GSEA) was used to analyze the enriched signaling pathways.

### Statistics

The data obtained was analyzed using Statistical Product and Service Solution (SPSS) software (Version 20.0, IBM). The result of viral multiplication, the expression levels of immune-related genes in mIgM^+^ B lymphocytes and the rates of apoptotic B lymphocytes under different temperatures were analyzed by one-way analysis of variance (ANOVA). Values were considered as significant at *p* < 0.05.

## Results

### Infection Characteristics of HIRRV to mIgM^+^ B Lymphocytes

**Figure 1:**
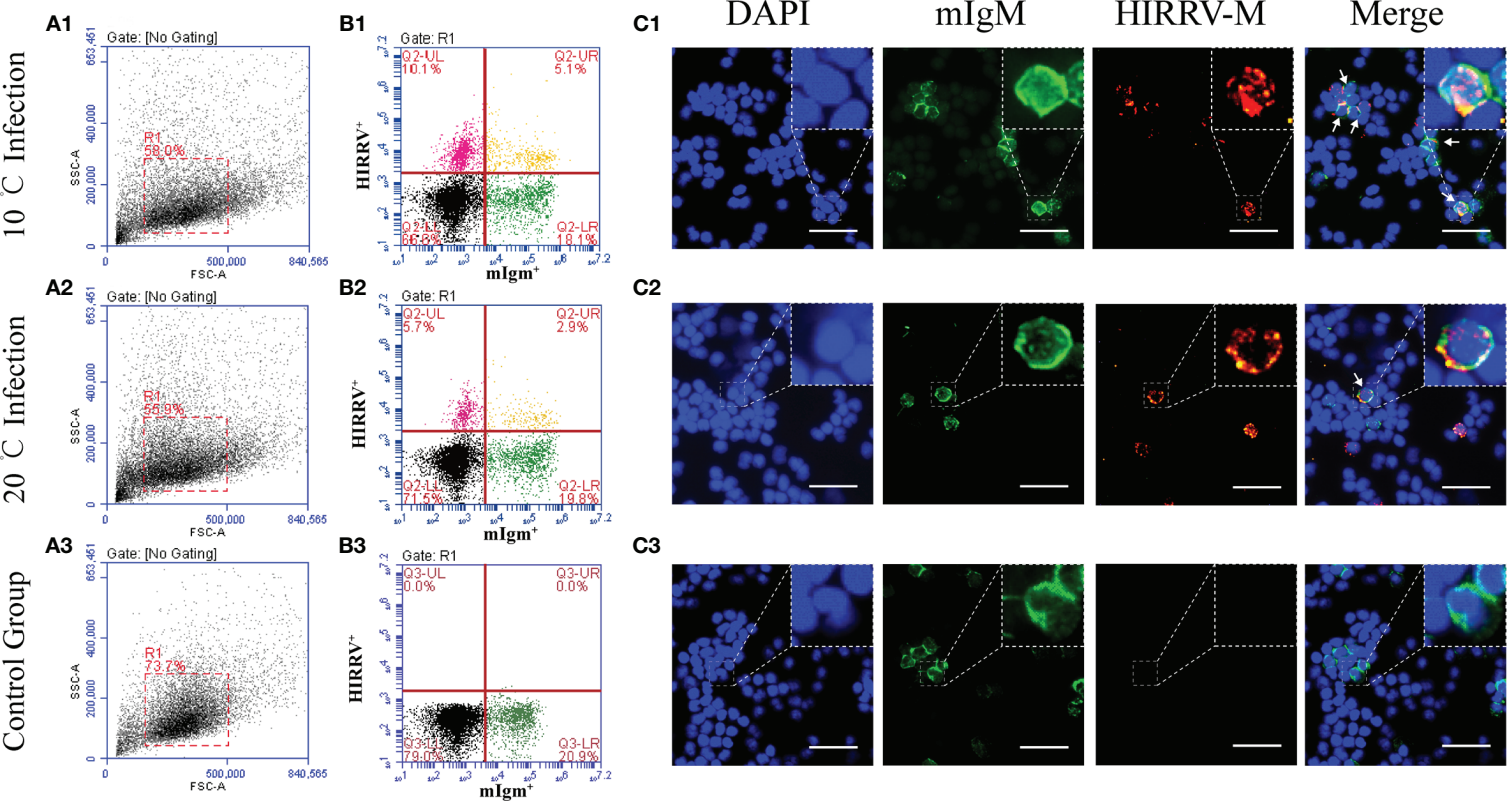
Infection characteristics of HIRRV to mIgM^+^ B lymphocytes at 10°C and 20°C. The proportions of HIRRV^+^/mIgM^+^ B lymphocytes (24 hpi) detected by flow cytometry and IIFA. **(A)** Flow cytometry results of B lymphocytes in peripheral blood leukocytes. FSC area (FSC-A)/SSC area (SSC-A) analyses are shown in **(A1–A3)**, and the red gate represents leukocytes. **(B)** HIRRV^+^/mIgM^+^ B lymphocytes. Mouse anti-flounder IgM mAbs and Mouse anti-HIRRV M protein mAbs were used in this experiment. **(C)** Immunofluorescence staining results of HIRRV^+^/mIgM^+^ B lymphocytes. (1), 10°C infection; (2), 20°C infection; (3), Control Group. Arrows indicate HIRRV^+^/mIgM^+^ B lymphocytes, Bar = 20 μm.

**Figure 2:**
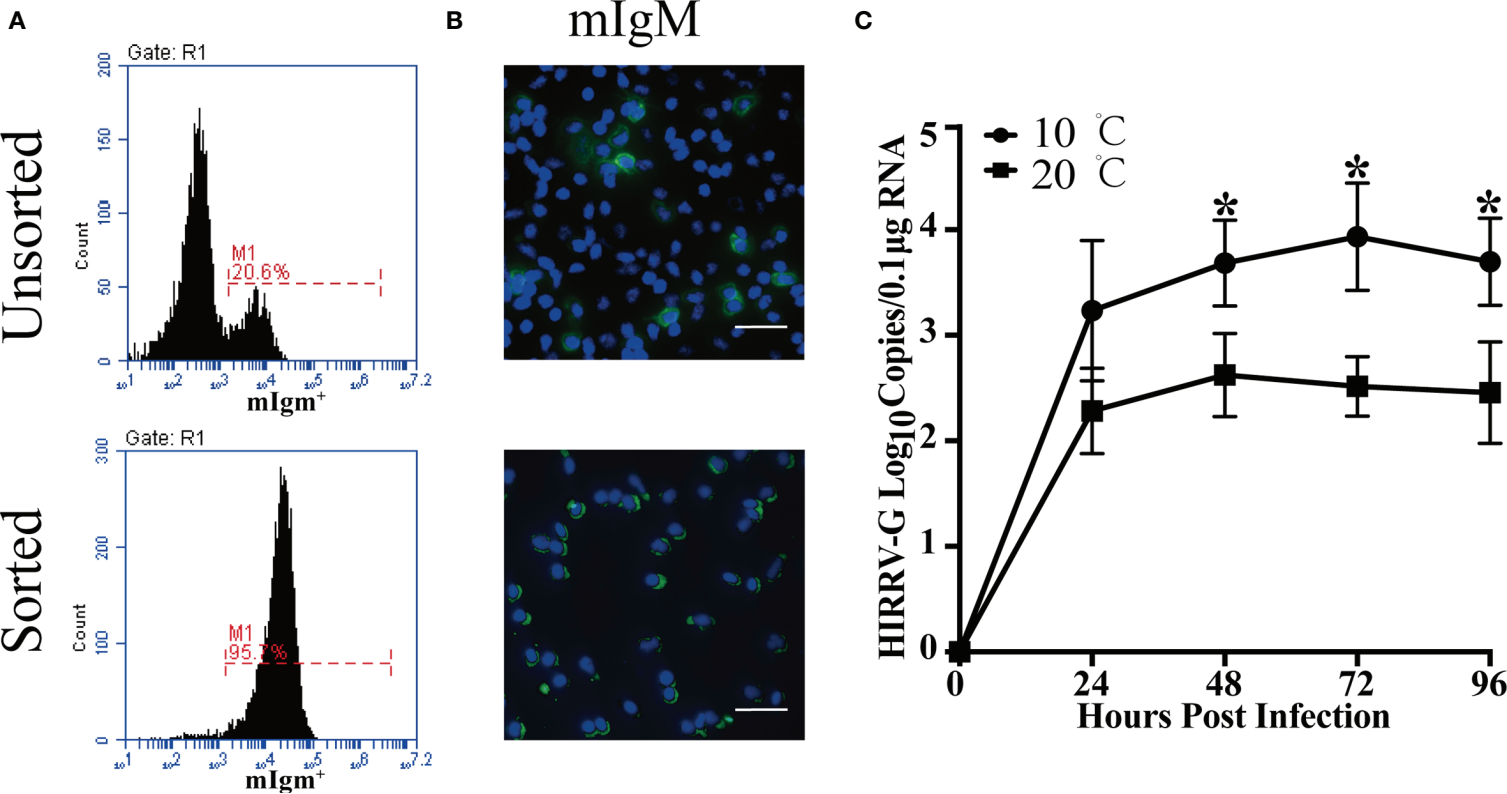
Detection of HIRRV replication in the sorted mIgM^+^ B lymphocytes. **(A)** Flow cytometry results of unsorted and sorted mIgM^+^ B lymphocytes from PBLs (24 hpi). **(B)** Immunofluorescence staining results of unsorted and sorted HIRRV^+^/mIgM^+^ B lymphocytes. Bar = 20 μm. Mouse anti-flounder IgM mAbs were used in this experienment. **(C)** The changes of viral copy numbers in mIgM^+^ B lymphocyte of HIRRV infected flounder maintained at 10°C and 20°C. Mean viral copy numbers and standard deviation were represented in log_10_ scale. The results were presented as the means ± SEM of three individuals, asterisk indicate significant difference between 10°C and 20°C infection groups (*p* < 0.05).

The proportions of HIRRV^+^/mIgM^+^ B lymphocytes in PBLs of viral infected flounder maintained at 10°C and 20°C were analyzed by flow cytometry. In the HIRRV infected flounder maintained at 10°C, the proportion of HIRRV^+^/mIgM^+^ B lymphocytes in PBLs was 5.1% at 24 hpi, which accounts for 22.0% of total mIgM^+^ B lymphocytes, and 10.1% HIRRV^+^/mIgM^-^ cells were also found in PBLs. In the HIRRV infected flounder maintained at 20°C, the proportion of HIRRV^+^/mIgM^+^ B lymphocytes in PBLs was 2.9% at 24 hpi, which accounts for 12.8% of total mIgM^+^ B lymphocytes, and 5.7% HIRRV^+^/mIgM^-^ cells were also found in PBLs. In flounder injected with PBS, no HIRRV^+^ cell was detected in PBLs (**Figure 1**). IIFA showed that HIRRV^+^/mIgM^+^ B lymphocytes with red and green fluorescence could be observed in the PBLs from HIRRV infected flounder maintained at 10°C and 20°C, and HIRRV^+^/mIgM^-^ cells were also observed only with red fluorescence signal located mainly around membrane There was no HIRRV positive signal in PBLs from flounder injected with PBS (**Figure 1**). By IMB method, high purity (95.7%) of mIgM^+^ B lymphocytes was sorted from PBLs with 20.6% mIgM^+^ B lymphocytes (**Figure 2A**). IIFA showed that some lymphocytes had green fluorescence signal on the cell membrane before sorting, while all sorted mIgM^+^ B lymphocytes had green fluorescence signal on the cell membrane, indicating high-purity mIgM+ B lymphocytes were sorted (**Figure 2B**). The changes of viral copies in mIgM^+^ B lymphocytes sorted from HIRRV infected flounder maintained at 10°C and 20°C were detected by qRT-PCR. The viral copies were significantly increased at 24 hpi with 10^3.2^ and 10^2.3^ copies/0.1 μg RNA in 10°C and 20°C infection groups, respectively. There was no significant difference in viral copies of two infection groups from 24 hpi to 96 hpi. However, the viral copies in 10°C infection group were significantly higher than that in 20°C infection group at 48, 72 and 96 hpi (**Figure 2C**).

### Differential Gene Expression Profiles in the mIgM^+^ B Lymphocytes of Uninfected Flounder Between 10°C and 20°C

**Figure 3:**
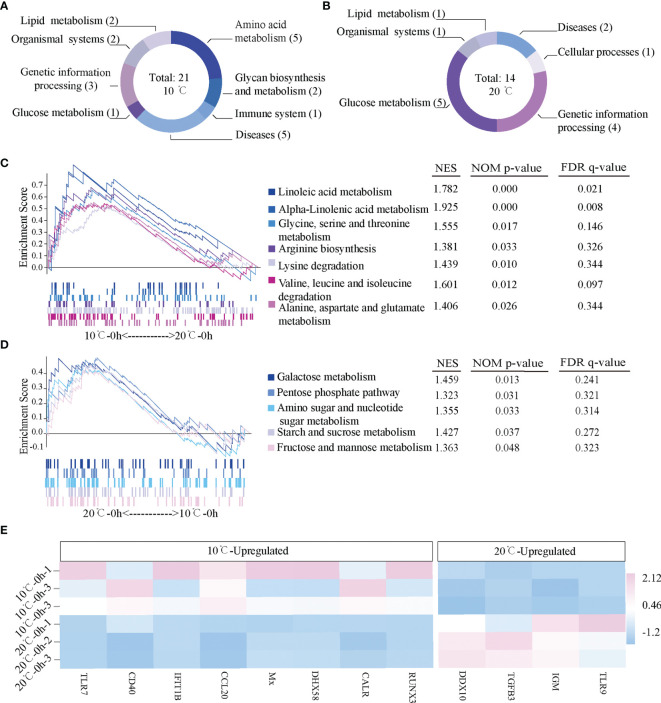
Gene expression profiles in the mIgM^+^ B lymphocytes of uninfected flounder maintained at 10°C and 20°C. **(A, B)** The activated signal pathways at 10°C and 20°C. **(C, D)** Metabolic pathways activated in 10°C and 20°C. Normalized enrichment score (NES) and values of the pathways with *p* < 0.05 are presented. **(E)** The heatmap of immune related genes at 10°C and 20°C.

The difference of active signal pathways in the mIgM^+^ B lymphocytes of uninfected flounder between 10°C and 20°C was analyzed by GSEA. There were 21 active signal pathways at 10°C (**Figure 3A**), and 14 active signal pathways at 20°C (**Figure 3B**). The metabolic pathways in mIgM^+^ B lymphocytes differs markedly between 10°C and 20°C, Lipid metabolism and Amino acid metabolism pathways were significantly enhanced at 10°C, including Linoleic acid metabolism, alpha-Linolenic acid metabolism, Glycine, serine and threonine metabolism, Arginine biosynthesis, Lysine degradation, Valine, leucine and isoleucine degradation, and Alanine, aspartate and glutamate metabolism (**Figure 3C**). In contrast, Glucose metabolism were significantly enhanced in mIgM^+^ B lymphocytes of uninfected flounder at 20°C, including Galactose metabolism pathway, Pentose phosphate pathway, Amino sugar and nucleotide sugar metabolism, Starch and sucrose metabolism and Fructose and mannose metabolism (**Figure 3D**). Although the differential expression genes between 10°C and 20°C were mainly enriched in metabolic pathways, some immune related genes had significantly different levels between two uninfected groups. Significantly higher expression levels of *IGM*, Toll-like recptor-9 (*TLR9*), survivin-transforming growth factor beta 3 (*TGFB3*) and DEAD box helicase 10 (*DDX10*) were detected at 20°C, while Runt-related transcription factor 3 (*RUNX3*), Calreticulin (*CALR*), DExH-Box helicase 58 (*DHX58*), Myxovirus resistance (*Mx*), Chemokine Ligand 20 (*CCL20*), Interferon-induced with tetratricopeptide repeats 1B (*IFIT1B*), *CD40* and Toll-like recptor-7 (*TLR7*) were significantly up-regulated at 10°C (**Figure 3E**).

### Immune Response of mIgM^+^ B Lymphocytes to HIRRV Infection at 10°C and 20°C

**Figure 4:**
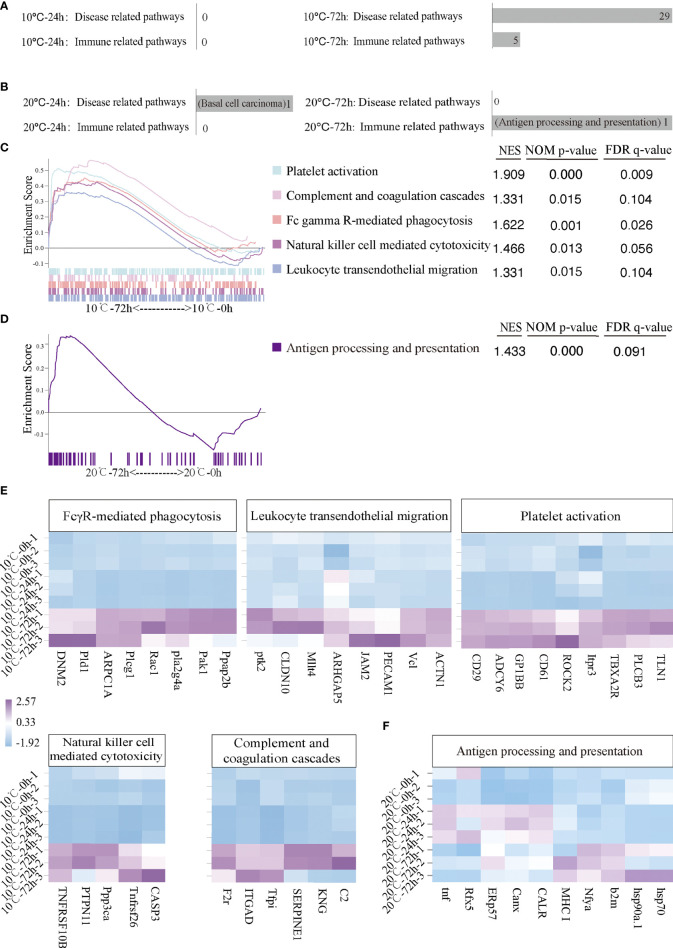
Activated immune related and disease related pathways and related genes of infected flounder maintained at 10°C and 20°C. **(A, B)** The number of activated disease-related and immune-related pathways at 10°C and 20°C. **(C, D)** The activated immune-related pathways in 10°C-72 hpi group and 20°C-72 hpi group. NES values of the pathways with *p* < 0.05 are presented. **(E, F)** The heatmap of immune genes in each activated immune-related pathway at 10°C and 20°C.

The signal pathways involved in immune response in mIgM^+^ B lymphocytes of infected flounder at 10°C and 20°C were analyzed by GSEA. At 10°C, no disease-related or immune-related signal pathway was significantly activated at 24 hpi, but 29 disease-related and five immune related signal pathways were significantly activated at 72 hpi (**Figure 4A**). The five immune related signal pathways include Platelet activation, Complement and coagulation cascades, FcγR-mediated phagocytosis, Natural killer cell mediated cytotoxicity and Leukocyte transendothelial migration (**Figure 4C**). Furthermore, the immune genes up-regulated in each pathway were listed by heatmap (**Figure 4E**). Specifically, PhosPhatidic Acid Phosphatase type 2B (*Ppap2b*), P21-activated kinase 1 (*Pak1*), Phospholipase A2-IVA (*Pla2g4a*), Ras-related C3 botulinum toxin substrate 1 (*Rac1*), Phospholipase C gamma 1 (*Plcg1*), Actin-related protein 2/3 complex subunit 1A (*ARPC1A*), Phospholipase D 1 (*Pld1*) and Dynamin 2 (*DNM2*) were significantly up-regulated in FcγR-mediated phagocytosis pathway; alpha-actinin 1 (*ACTN1*), Vinculin (*Vcl*), Platelet endothelial adhesion molecule 1 (*PECAM1*), Junction adhesion molecule 2 (*JAM2*), Lymphoid or mixed-lineage leukemia; translocated to, 4 (*Mllt4*), Claudin 10 (*CLDN10*) and Protein tyrosine kinase 2 (*Ptk2*) were significantly up-regulated in Leukocyte transendothelial migration pathway; Talin-1 (*TLN1*), Phospholipase C-beta 3 (*PLCB3*), Thromboxane A2 receptor (*TBXA2R*), Inositol 1,4,5-trisphosphate receptor type 3 (*Itpr3*), Rho-associated protein kinase 2 (*ROCK2*), Integrin beta 3 (*CD61*), Glycoprotein Ib platelet subunit beta (*GP1BB*), Adenylate cyclase type 6 (*ADCY6*) and Integrin beta 1 (*CD29*) were significantly up-regulated in Platelet activation pathway; Caspase-3 (*CASP3*), TNF receptor superfamily 26 (*Tnfrsf26*), Protein phosphatase 3 catalytic subunit alpha isoform (*Ppp3ca*), Protein Tyrosine Phosphatase Non-Receptor Type 11 (*PTPN11*) and TNF-receptor superfamily member 10b (*TNFRSF10B*) were significantly up-regulated in Natural killer cell mediated cytotoxicity pathway; Complement Component 2 (*C2*), Kininogen (*KNG*), Serine protease inhibitor clade E member 1 (*SERPINE1*), Tissue factor pathway inhibitor (*Tfpi*), Integrin alpha D (*ITGAD*) and Coagulation factor II receptor (*F2r*) were significantly up-regulated in Complement and coagulation cascades pathway. At 20°C, one disease-related signal pathway namely Basal cell carcinoma was significantly activated at 24 hpi but no immune related signal pathway was significantly activated (**Figure 4B**). In contrast, no disease-related signal pathway was significantly activated at 72 hpi but one immune related signal pathway, namely Antigen processing and presentation pathway, was significantly activated (**Figure 4D**). Specifically, *HSP70, HSP90a.1*, Beta (2) -microglobulin (*B2M*), Nuclear transcription factor Y, subunit A (*Nfya*), Major histocompatibility complex 1 (*MHC1*)*, CALR*, Calnexin (*Canx*)*, ERp57*, Regulatory factor x 5 (*Rfx5*) and Tumor necrosis factor (*TNF*) were significantly up-regulated in Antigen processing and presentation pathway (**Figure 4F**).

### Longitudinal Changes of Immune-Related Pathways in mIgM^+^ B Lymphocytes of Infected Flounder at 10°C and 20°C

**Figure 5:**
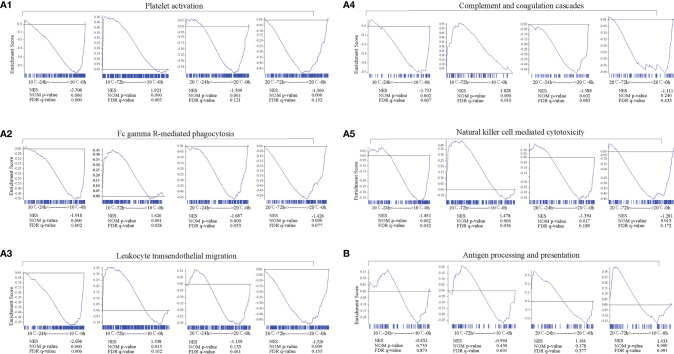
Longitudinal changes of immune-related pathways in mIgM^+^ B lymphocytes of infected flounder at 10°C and 20°C. **(A1-A5, B)** The immune-related pathways including Platelet activation **(A1)**, FcγR-mediated phagocytosis **(A2)**, Leukocyte transendothelial migration **(A3)**, Complement and coagulation cascades **(A4)**, Natural killer cell mediated cytotoxicity **(A5)**, and Antigen processing and presentation **(B)** enriched in samples. GSEA was used to analyze the signaling pathways enrichment in different groups. Normalized enrichment score (NES) indicated the analysis results across gene sets. False discovery rate (FDR) presented if a set was significantly enriched. ES, enrichment score.

The longitudinal changes of immune-related pathways including Platelet activation, Complement and coagulation cascades, FcγR-mediated phagocytosis, Natural killer cell mediated cytotoxicity, Leukocyte transendothelial migration and Antigen processing and presentation were similar at low temperature but slightly different at high temperature. At 10°C, Platelet activation, Complement and coagulation cascades, FcγR-mediated phagocytosis, Natural killer cell mediated cytotoxicity and Leukocyte transendothelial migration were significantly down-regulated at 24 hpi (*p* < 0.05) but activated at 72 hpi (*p* < 0.05), while the Antigen processing and presentation pathway was slightly down-regulated but without significant difference both at 24 and 72 hpi (*p* > 0.05). At 20°C, except for the Antigen processing and presentation pathway, the other pathway mentioned above were almost all significantly down-regulated at 24 and 72 hpi (*p* < 0.05) (**Figure 5A**). In contrast, the Antigen processing and presentation was activated at 20°C and showed a significantly up-regulation at 72 hpi (*p* < 0.05) (**Figure 5B**).

### Temporal Expressions of Immune- Related Genes and Key Genes in Antigen Processing and Presentation Signal Pathway

**Figure 6:**
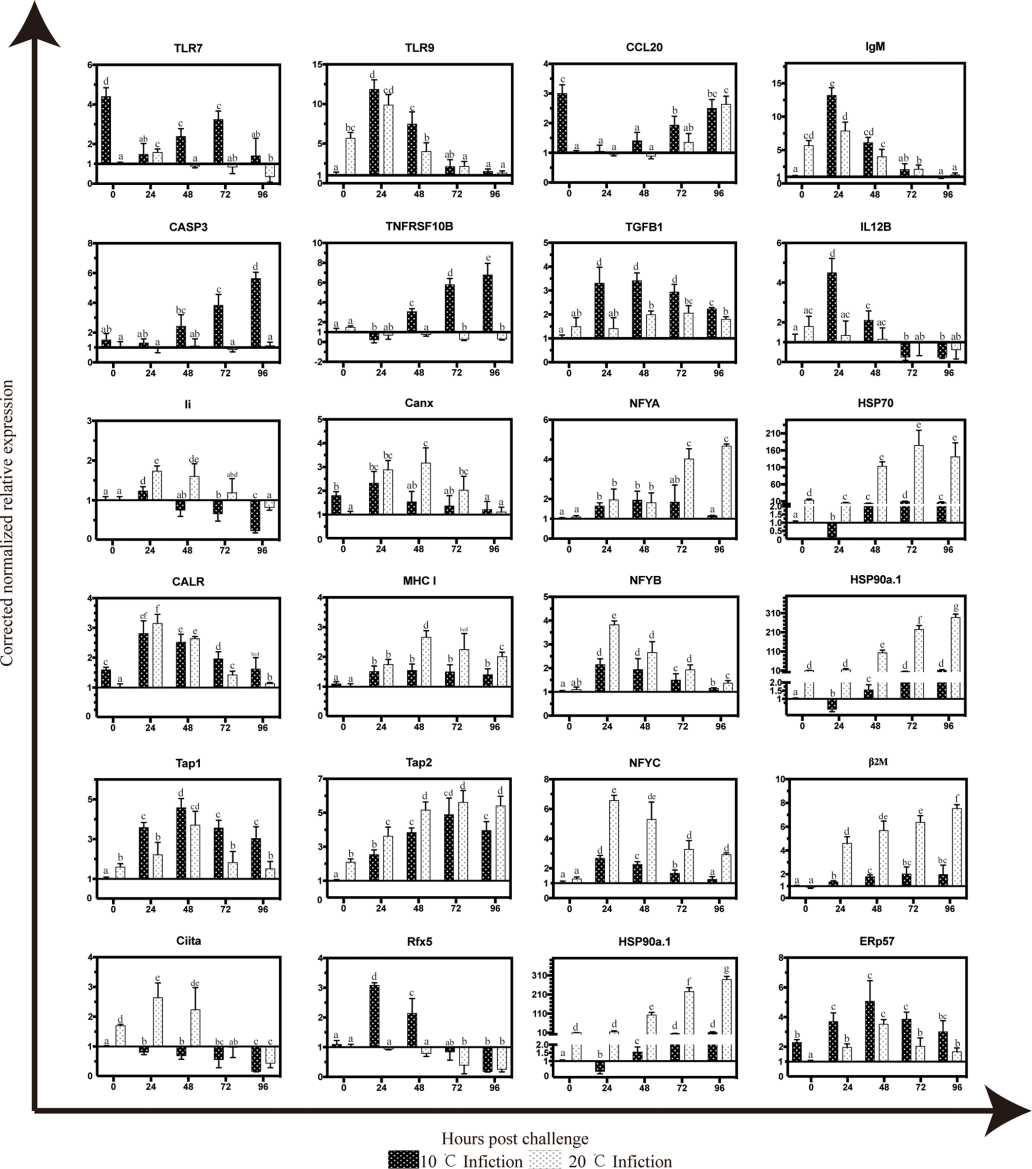
Temporal expression patterns of selected gene targets in mIgM^+^ B lymphocytes of infected flounder as assessed by qPCR. The results were presented as the means ± SEM of three individuals, different letters above the error bars indicate significant differences (*p* < 0.05).

The temporal expression of several key genes in Antigen processing and presentation pathway were detected by qRT-PCR, including nuclear transcription factor Y (*NFYA*,*NFYB* and *NFYC*), two with heat shock protein (*HSP70* and *HSP90a.1*), two relating to encoding transporters Transporter associated with antigen processing 1 (*TAP1*) and Transporter associated with antigen processing 2 (*TAP2*), two transcription and translation regulators *Ciita* (Class II trans-activator) and *Rfx5*, three involved in major histocompatibility complex *β2M*, HLA class II histocompatibility antigen gamma chain/CD74 (*Li*), and *MHC I*, endoplasmic reticulum chaperones (*CALR* and *Canx*), *TNF*, several genes associated with inflammatory response (*TLR7, TLR9, CCL20* and *IgM*) and apoptosis related genes (*TNFRSF10B* and *CASP3*). The expression of *TAP1, TAP2*, and *Rfx5* increased with time only at 10°C. At 20°C, the expression of *Ciita*, *NFYB, NFYC*, *CALR, Canx* and *TNF* reached the peak at 24 hpi, which was significantly higher and earlier compared to 10°C infection group. *HSP90a.1* and *β2M* showed stronger response at 20°C and continued to rise after infection and the expression levels of them were significantly higher than those in 10°C infection group. The expression of *TLR9 and IgM* decreaced with time both in 10 and 20°C, *TLR7* and *CCL20* have increased since 24 hpi. Only at 10°C, the expression of *TNFRSF10B* and *CASP3* increased with time and reached the peak at 72 hpi. (**Figure 6**).

### The Apoptotic Response of mIgM+ B Lymphocytes to HIRRV Infection

**Figure 7:**
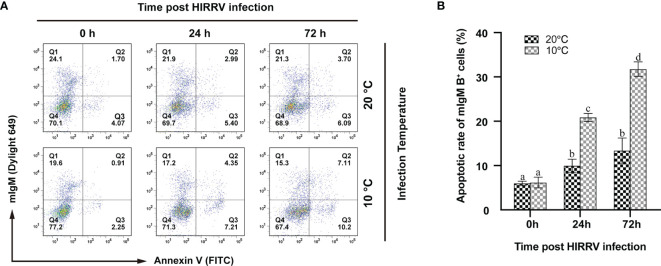
The changes of apoptotic lymphocytes post HIRRV infection by flow cytometry. **(A)** The fluorescence dot plots represent apoptotic and/or mIgM^+^ B lymphocytes sampled at 0, 24, 72 hpi at 10 and 20°C, respectively. **(B)** Apoptotic rates of mIgM^+^ B lymphocytes. The results were presented as the means ± SEM of three individuals, different letters above the error bars indicate significant differences (*p* < 0.05).

According to the results of flow cytometry, the rates of apoptotic mIgM^+^ B lymphocytes showed a significant increase after HIRRV infection both at 10°C and 20°C. At 10°C, the percentage of apoptotic lymphocytes was 20.89 ± 0.90% at 24 hpi and significantly increased to 31.85 ± 1.63% at 72 hpi (*p* < 0.05). In contrast, the apoptotic rates of mIgM^+^ B lymphocytes at 20°C were significantly lower than that at 10°C. At 20°C, the apoptotic rates reached the values of 9.97 ± 1.47% and 13.38 ± 2.83% at 24 and 72 hpi, respectively, however, there was no significant difference between these two time points (**Figure 7**).

## Discussion

In this study, the protein and nucleic acid of HIRRV were detected in some mIgM^+^ B lymphocytes from the viral infected flounders at 24 hpi, indicated that some mIgM^+^ B lymphocytes were susceptible to HIRRV. HIRRV-M protein and HIRRV-G gene were also detected in mIgM^+^ B lymphocytes, which further demonstrated that HIRRV could infect mIgM^+^ B lymphocytes. In mammalian,RNA viruses such as Epstein Barr virus (EBV) (41), Bovine respiratory syncytial virus (BRSV) (42), Measles virus (MV) (43) could infect B lymphocytes and affect the pathogenicity of virus and host immunity in different ways. In the study of rhabdovirus, live RABV-based vaccine vectors efficiently infect naive primary murine and human B cells ex *vivo* (44), VHSV have the capacity to infect rainbow trout spleen IgM^+^ B lymphocytes (37). However, not all B lymphocytes were susceptible to virus in these studies, the same phenomenon was observed here. One possible explanation is that the mIgM^+^ B lymphocytes at different developmental stages might have different susceptibility to HIRRV. Studies on MV show that the sensitivity of MV to different human B lymphocyte subsets is different, and immature and memory B lymphocytes are more susceptible to MV (43). However, the identification of B lymphocytes in different stages of flounder cannot be performed due to the lack of specific probes at present, which needs further study in the future. In addition, the positive signal of HIRRV-M was also detected to be located around some mIgM^-^ lymphocyte membrane which is consistent with the research result of HIRRV-infected EPC cells (38). The M protein is involved in the entry of rhabdovirus into its host cell and in the assembly and budding of new viral particle at the cell surface, the M protein spontaneously associates with the cytoplasmic membrane, where it recruits newly synthesized nucleocapsids (45); the sequence of the M protein contains a motif of interaction with the host cell machinery involved in multi-vesicular body (MVB) formation and it mediates the release of viral particles by redirecting this machinery to the cytoplasmic membrane (46, 47). Therefore, it can be speculated that HIRRV could infect some mIgM^-^ lymphocytes, but the specific lymphocyte subsets which are susceptible to HIRRV in PBLs were not clear. Current research on RNA virus infecting lymphocytes mainly focuses on mammals. For example, studies have shown that Ebola virus (EBOV) could infect human T lymphocytes and cause abortive infection, which eventually leads to endoplasmic reticulum stress-induced autophagy and subsequent cell death (48). However, whether T lymphocytes of flounder were susceptible to HIRRV remains to be further confirmed.

This study also found that there was no significant difference in the viral copy numbers from 24 to 96 hpi both at 10°C or 20°C, indicated that the replication of HIRRV in mIgM^+^ B lymphocytes was inhibited by some factors. Similarly, it was found that VHSV could not proliferate efficiently in mIgM^+^ B lymphocytes (37). Viral infection is an intricate and complex process, including entrying host cells, viral genome replication and transcription, viral protein synthesis, viral assembly and release. Even if the virus can smoothly enter the cell, any link problems will lead to infection failure. Nevertheless, the viral copy numbers in mIgM^+^ B lymphocytes at 10°C were higher than that at 20°C, the same phenomenon also appeared in the spleen of flounder infected with HIRRV (4), indicating that the mIgM^+^ B lymphocytes at low temperature were more susceptible to HIRRV infection. The occurrence of disease is the result of the interaction between virus and host, both of which are affected by the change of temperature. Previous studies on Salmon alpha virus (SAV) have shown that high temperature (27°C) can cause viral glycoprotein E2 to misfold, making it unable to be processed into mature glycoproteins. Therefore, low temperature is conducive to the replication of SAV (49). Studies on zebrafish (*Danio rerio*) and grouper (*Epinephelus septemfasciatus*) showed that low temperature can significantly inhibit the expression of some antiviral genes, such as TLR3, IFN-γ and Mx (50, 51). It was also shown that the phagocytic ability, antigen presentation ability and antibody secretion level of mIgM^+^ B lymphocytes of teleost are generally inhibited at low temperature (20, 52, 53). Therefore, the damage of immune function of mIgM^+^ B lymphocytes at low temperature may also be an important factor for the increase of HIRRV copy number. On the other hand, the viral copy numbers of HIRRV in mIgM^+^ B lymphocytes were significantly lower than that in spleen of flounder (4), indicating that there is a mechanism to inhibit the replication of HIRRV in mIgM^+^ B lymphocytes. Therefore, a further study is needed to explore the responses of mIgM^+^ B lymphocytes to HIRRV infection.

In order to clarify the effect of temperature on mIgM^+^ B lymphocytes, this study used transcriptome to perform data mining on mIgM^+^ B lymphocytes from uninfected flounders at 10°C and 20°C. GSEA analysis showed that the pathways related with Amino acid metabolism and Lipid metabolism were up-regulated at 10°C compared with 20°C, such as Lysine degradation, Alpha-Linoleic acid metabolism and Linoleic acid metabolism. It is generally believed that Amino acid metabolism and Lipid metabolism play a key role in generating energy to resist low temperature. When stimulated by low temperature, protein will decompose into amino acids which are then may utilized for anti-stress protein synthesis and turnover (54). The metabolism of fatty acids in fish is sensitive to temperature. Cold stress will reduce the proportion of saturated fatty acids in cells (55) and increase the proportion of unsaturated fatty acids (56), which is conducive to maintaining cell membrane fluidity. Previous research has also shown increases in unsaturated fatty acids in the plasma membrane have also been observed in mIgM^+^ B lymphocytes from fish at low temperature but the effect of this change on lymphocyte function has not been elucidated, the authors speculated that it stiffened cell membranes, decreasing cell-to-cell interactions (17). Moreover,viral replication is tightly linked to the host cell metabolism, study of snakehead fish vesiculovirus (SHVV) revealed that glutamine was required for SHVV propagation, glutamine deprivation significantly reduced the expression of the mRNAs and proteins of SHVV, and the production of virus particles (57). On the other hand, metabolism is able to affect the immunity of the host cell. For example, activation of the pentose phosphate pathway would lead to NADPH production. NADPH is used by the NADPH oxidase to generate reactive oxygen species (ROS) during the respiratory burst, and ROS production would increase the expression of anti-inflammatory genes, which in turn prevent excessive tissue damage (58, 59). In the present study, the differential expression of glutamate metabolism and pentose phosphate pathway at 10°C and 20°C might affect the outcome of interaction between HIRRV and mIgM^+^ B lymphocytes. According to the results of GSEA, the differences in the expression of mIgM^+^ B lymphocytes mainly focused on the metabolism-related signal pathways between 10°C and 20°C while the difference in immune-related signal pathways was less. In these upregulated immune genes, the expression of *CCL20* gene deserve attention. *CCL20* is a cysteine–cysteine chemokine that was originally shown to be chemotactic for immature dendritic cells, effector or memory CD4^+^ T lymphocytes, and B lymphocytes. Results of several studies demonstrate enhanced proinflammatory cytokine expression from immune cells cooled *in vitro* (60, 61). Chemokines are key mediators of leukocyte recruitment during immunoregulatory and proinflammatory responses. It indicates that low temperature can cause inflammatory reaction in flounder. On the other hand, *IgM* has been significantly upregulated at 20°C. IgM is distributed on the surface of B cells and is responsible for antigen recognition, antigen endocytosis and extracellular signal transmission (62). IgM is the most classical immune marker in the upregulated immune genes at 20°C. Similar results were also found in rainbow trout, in which an increase in transcript expression was observed at 15°C for B cell marker-IgM in the anterior kidney post-infection with *Tetracapsuloides bryosalmonae*, however, no significant up-regulation was observed at 12°C, indicating that B cell activation in response to pathogens may be impaired at low temperatures (18). It is reasonable to speculate that the upregulation of IgM expression at 20°C may be one of the reasons for the inhibition of HIRRV in flounder. Meanwhile, the decreased expression of IgM at 10°C suggests that several IgM-mediated signal pathways were inhibited at low temperature.

Activation of the Complement and coagulation cascades, FcγR-mediated phagocytosis, Platelets activation, Leukocyte transendothelial migration and Natural killer cell mediated cytotoxicity pathways have been observed in mIgM^+^ B lymphocytes at 72 hpi under 10°C. Complement cascade and blood coagulation are tightly interwoven and two major contributors to the first line of defense against infection, activated complements have shown inhibition activity against viral infections such as influenza virus (63–65). The activation of coagulation cascade during viral infection may be considered as a protective response and to limit the spread of pathogens (66). However, excessive activation of the coagulation cascade can be deleterious. It is worth noting that *F2R* (coagulation factor II receptor) gene was involved in the Complement and coagulation cascades signal pathway, *F2R* also known as *PAR1* (Protease-activated receptor 1) that contributes to inflammatory responses. Studies indicated that *PAR1* activation increased inflammation, early virus production, weight loss, and mortality after infection (67), and the activation of *PAR1* increases HSV infection of endothelial cells *in vitro* (68). In the present study, the expressions of *F2R* (*PAR1*) were upregulated and the specific function in HIRRV infection still needs further studies. Our previous study has shown that mIgM^+^ B lymphocytes of flounder possessed potent phagocytic activity and can phagocytize fluorescent microspheres and bacteria (69). Phagocytosis is an essential process of the innate immune response, which can be initiated by FcγR in membranes, enabling immune cells to eliminate invasive pathogens by internalizing them in phagosomes (70).Phagosome formation, as well as maturation, involves membrane trafficking between multiple intracellular membrane compartments. Classical actors of the exocytotic process such as *PLD1* participate in these trafficking events (71).The activation of FcγR-mediated phagocytosis pathway and up-regulation of *PLD1*, *Pla2g4a*, *Ppap2b* gene expression was found in 10°C infection group, suggesting that the phagocytosis of mIgM^+^ B lymphocytes could be activated at low temperature during HIRRV infection. Notable among these genes was *PLD1*. Studies demonstrate that virus infection stimulates *PLD* activity and *PLD* could facilitate the rapid endocytosis of influenza virus, permitting viral escape from innate immune detection (72). Lipid species playing structural roles in viral entry and budding, the viral surface protein binding to target cell surface, triggering endocytosis, allowing the virus entry inside the host cell. After viral proteins are synthesized, they accumulate at the cell membrane, complex with viral genomes, and bud off the cell surface, forming the viral envelope from the host cell membrane (73). Some virus like Influenza viruses (72) and HIV (74) exploit these fundamental processes within the host cell to facilitate entry and subsequently drive virus replication, this may be one way for HIRRV to entry into mIgM^+^ B lymphocytes. In addition to the phagocytosis function of mIgM^+^ B lymphocytes, platelets also play crucial role in antiviral immunity, such as immune surveillance, inflammation and host defence during infection (75). In the activated platelet activation pathway, *CD29* (76) *CD61* (77) can mediate the adhesion of plates and lymphocytes, and promote physical interaction facilitated by a variety of receptors, resulting in the increase of platelet leutocyte aggregates (PLAs) (78). Importantly, platelet-leukocyte interactions facilitate leukocyte recruitment and extravasation to sites of inflammation (79, 80), suppressing the production of pro-inflammatory cytokines and augmenting the expression of the anti-inflammatory cytokine (81, 82), which was also observed after infection at 10°C in the present experiments. A significant decrease in representative pro-inflammatory factor (*IL12B*) expression with prolonged infection was observed while anti-inflammatory transforming growth factor beta-1 (*TGF-β1*) expression was continuously high during infection at 10°C. *TGF-β1* has anti-inflammatory and immune suppressive characteristics manifested in suppression of differentiation of Th cells type I and II thereby controlling inflammatory processes (83). The augment of *TGF-β1* may protect against excessive tissue damage induced by HIRRV.

Cellular cytotoxicity is an important effector mechanism of immune system to combat viral infections. In Natural killer cell mediated cytotoxicity pathway, TNF acts both directly on virus-infected cells as a cell death-inducing cytokine. TNF is well known as a critical factor in eliciting rapid inflammatory events acting through receptors (84). In general, ligation of these receptors results in activation of caspases, E3: ubiquitin ligases, or both. Apoptosis inducers activate zymogen caspases, and the subsequent signal cascade leads to the activation of CASP3, which performs the final steps in causing host cell death (85, 86). An early and effective apoptotic response, with CASP3 as an important effector, can shut down viral replication and disease development in the host (87). In our study, we found that the mRNA expression of *TNFRSF10B* and *CASP3* was significantly increased in the infected flounders at 72 hpi, and the apoptotic rates of mIgM^+^ B lymphocytes were also found to be significantly increased, suggesting the possibility of mIgM^+^ B lymphocytes relying on apoptosis to combat viral infections at low temperature. Based on the above evidence, it is suggested that the antiviral strategies of mIgM^+^ B lymphocytes at low temperature is inseparable from the joint action of multiple immune functions including activation of the Complement and coagulation cascades, enhancement of the phagocytic activity, recruitment and activation of inflammatory cells, inducing inflammatory and apoptosis responses.

The mortality of HIRRV infected flounder rarely occurred at 20°C, given such a scenario, the upregulated immune pathways in mIgM^+^ B lymphocytes at 20°C may be more important. Based on the GSEA, Antigen processing and presentation pathway as the only significantly up-regulated immune pathway, suggesting that this pathway may be the key for antiviral function. During the Antigen processing and presentation process, viral proteins in the cytoplasm are digested by proteasome to form small molecular peptides, which are bound to HSP70/90 in the cytoplasm, and then transported to endoplasmic reticulum by antigen peptide transporter (TAP) to process and modify into antigen peptides. MHC I molecules are synthesized in endoplasmic reticulum and bind with antigen peptide to form antigen peptide MHC I molecular complex. However, the heavy chain of MHC I is unstable and needs to be stabilized by binding with subunit β2M. The complex was transferred into Golgi apparatus and then transported to the surface of APCs by secretory vesicles for recognition and binding by corresponding CD8^+^ T lymphocytes and nature killer cells. According to the mIgM^+^ B lymphocytes transcriptome data, *HSP90a.1* and *β2M* expression at 72 hpi were significantly higher at 20°C compared to 10°C, furthermore, *MHC I* had been up-regulated both at 72 hpi at 10°C and 20°C. Therefore, the antiviral effector functions of *HSP90a.1* and *β2M* warrant more attention. Heat shock proteins (HSPs) act as molecular chaperones inside cells, regulating conformational change, translocation, assembly and degradation of cellular proteins. HSPs is not only involved in cellular stress response like temperature, salinity and other stress factors, but also involved in the antigen processing and presentation machinery as chaperones for antigenic proteins and peptides (88). In virus-infected cells, although viral proteins could bind to HSPs to utilize the protein folding machinery, however, some HSP-bound viral proteins are degraded by proteasomes and presented to MHC I, leading to recognition of the infected cells by cytotoxic T lymphocytes (CTL) (89). Therefore, the upregulation of HSPs are important events in antiviral system. It has been reported that the expressions of *HSP90* was also upregulated in flounder infected with HIRRV at 20°C compared to 10°C (90). In this study, the up-regulated expression of *HSP90a. 1* may mediate efficient antigen uptake and strongly enhance the efficiency of antigen presentation to T lymphocytes. TAP1 and TAP2, members of the MDR/TAP subfamily of ATP-binding cassette transporters, encode heterodimeric molecule involved in endogenous antigen processing. In mammals, the TAP1 protein, together with TAP2, forms the TAP complex, which resides on the endoplasmic reticulum (ER) membrane and is responsible for the pumping of degraded cytosolic antigenic peptides across the ER into the membrane-bound compartment for association with MHC I molecules (83, 91, 92). In addition to its well-known function of antigen presentation in adaptive immunity, TAP1 also as a virus-inducible negative regulator of innate immunity. Current study found increased expression of TAP1 in human lung epithelial cells, THP-1 monocytes, HeLa cells, and Vero cells following virus infection, and overexpression of TAP1 enhanced virus replication such as influenza A virus, vesicular stomatitis virus, and human enterovirus 71 by inhibiting the virus-triggered activation of NF-κB signaling and the production of IFNs, IFN-stimulated genes, and proinflammatory cytokines (93). Thus, besides the role in activation of adaptive immune response, the upregulation of TAP1 and TAP2 expression following HIRRV infection at 10°C might also play an important role in viral replication. Beta-2-microglobulin (*β2M*) protein acts as a stabilizing scaffold of MHC I, the stability of the MHC I might be compromised in the absence of β2M. Studies have shown that expression of β2M was temperature sensitive, VHSV infection in the rainbow trout monocyte/macrophage cell line RTS11 increased the protein levels of β2M at 14°C, but failed to up-regulate the protein levels of β2M at 2°C, which may partly led to compromised immune responses against VHSV at low temperatures (20). β2M is also an essential component of the MHC I antigen presentation pathway, and its main function is forming a complex with MHC I to trigger CTL immunity. The viral peptides presented by MHC I are recognized by CD8^+^ T lymphocytes which proliferate and differentiate to become CTL that in turn recognize virus-infected cells and induce several cell death inducers of the apoptotic pathway (94). Earlier experiments in floundrs have revealed that the expression of *CD8* was significantly repressed at 15°C, however, it showed an up-regulated expression at 20°C (95). Therefore, it is speculated that the decrease of β2M could reduce the synthesis of MHC I molecular complex, causing the decrease of CD8^+^ T lymphocytes proliferation. Though viral infection induced the up-regulation of β2M and MHC I both at 10°C and 20°C, their magnitudes of up-regulation at 10°C were significantly lower than that at reduced at 20°C, indicating the cellular adaptive immunity was inhibited at 10°C, which would be beneficial for the HIRRV replication. When flounders were infected by HIRRV, the gene expression patterns in Antigen processing and presentation pathway in mIgM^+^ B lymphocytes were significantly different at 10°C and 20°C, suggesting that the activation of Antigen processing and presentation pathway might be temperature dependent. In other words, timely recognition and presentation of HIRRV antigen to T lymphocytes can lead to effective immune response, which may be the key to the antiviral function of mIgM^+^ B lymphocytes at 20°C, and also an important reason for the low mortality of HIRRV under high temperature.

In conclusion, this study demonstrated that HIRRV could infect mIgM^+^ B lymphocytes. Without HIRRV infection, the metabolic features in mIgM^+^ B cells were quite different under 10°C and 20°C. Lipid metabolism and Amino acid metabolism were significantly activated at 10°C, and Glucose metabolism was significantly activated at 20°C. HIRRV infection at 10°C results in the activations of the Complement and coagulation cascades, enhancement of the phagocytic activity, recruitment and activation of inflammatory cells and induced inflammatory responses. The effective activation of Antigen processing and presentation pathway might be an important mechanism for the low mortality of HIRRV infection at 20°C. This study provided a extensive data on gene transcriptional differences of mIgM^+^ B lymphocytes from the flounder infected with HIRRV under different temperatures, which provides new insights into the mechanism of temperature-regulated antiviral defense in flounder.

## Data Availability Statement

The datasets presented in this study can be found in online repositories. The names of the repository/repositories and accession number(s) can be found below: https://www.ncbi.nlm.nih.gov/, accession ID- SRR13300096-SRR13300113.

## Ethics Statement

The animal study was reviewed and approved by Guide for the Use of Experimental Animals of the Ocean University of China EU84 2010/63.

## Author Contributions

Designed the experiments: XT, XM, and WZ. Performed the experiments: XM and JC. Analyzed the data: XM, JC, and XT. Provided reagents/materials/analysis tools: JX, XS, and HC. Wrote the manuscript: XT, XM, and JC. All authors participated in the revision of the manuscript and confirmed the integrity of this work.

## Funding

This study was supported by the National Natural Science Foundation of China (31872590, 31730101, 31672685, 31672684, 31472295), Natural Science Foundation of Shandong Province (ZR2019MC029), the National Key Research and Development Program of China (2018YFD0900504) and Taishan Scholar Program of Shandong Province.

## Conflict of Interest

The authors declare that the research was conducted in the absence of any commercial or financial relationships that could be construed as a potential conflict of interest.

## Publisher’s Note

All claims expressed in this article are solely those of the authors and do not necessarily represent those of their affiliated organizations, or those of the publisher, the editors and the reviewers. Any product that may be evaluated in this article, or claim that may be made by its manufacturer, is not guaranteed or endorsed by the publisher.
